# Supplementary material for: Dual-Shell Microcapsules for High-Response Efficiency Self-Healing of Multi-Scale Damage in Waterborne Polymer–Cement Coatings
Source: Polymers (Basel). 2023 Dec 29;16(1):105. doi: 10.3390/polym16010105 (PMC10781181; doi:10.3390/polym16010105)
Supplement: Supplementary file 1 [file polymers-16-00105-s001.zip › polymers-2770272-Supporting Information-1st revised.pdf]

Supporting Information

Dual-shell microcapsules for high-response efficiency self-healing of multi-scale damage in waterborne polymer-cement coatings

Chenyang Liu[1], Zhicheng Sun[1]*, Shouzheng Jiao[2], Ting Wang[1], Yibin Liu[1], Xianyu Meng[3], Binbin Zhang[3], Lu Han[1], Ruping Liu[1], Yuanyuan Liu[4]， Yang Zhou[4]*

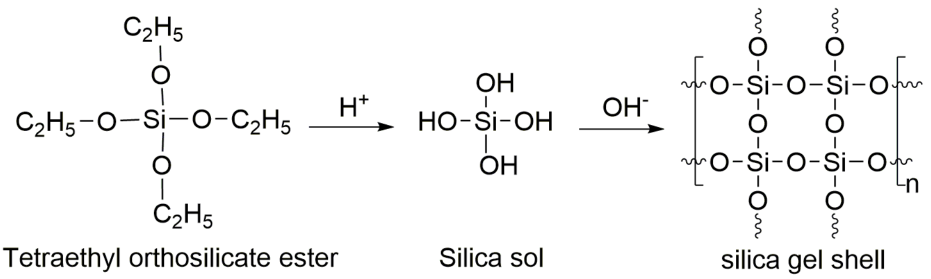

Figure S1 Reaction formula for silica gel shell preparation

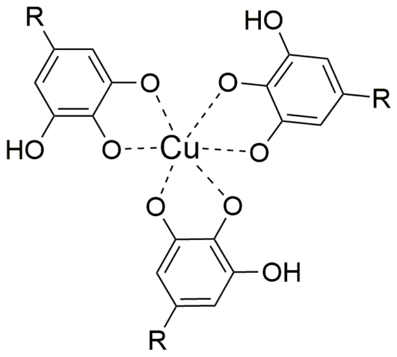

Figure S2 Structural formula of TA-Cu^{2+}chelates

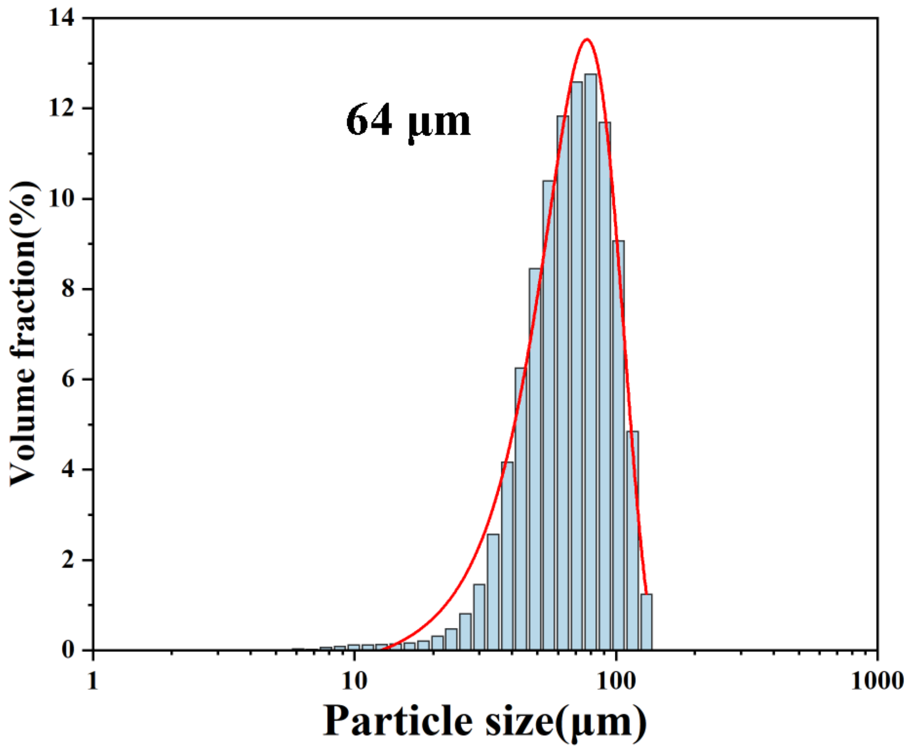

Figure S3 Particle size distributions of microcapsules

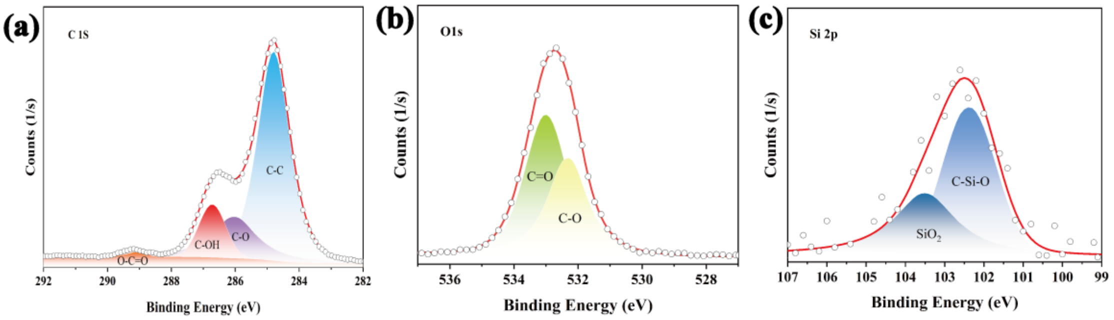

Figure S4. Self-healing microcapsules characterization by XPS analysis. (a) C1s peak was deconvoluted to 284.8, 286.7, 286 and 289 eV, corresponding to C-C, C-O-Ph, C-O-C/C-OH, and C=O functionalities in TA, respectively. (b) O1s spectrum was deconvoluted to 533.0 and 532.4 eV, representing the organic C-O and O-H structures, respectively. (c) The absorption peaks of Si2p at 102.3 and 103.5 are derived from C-Si-O and SiO2, respectively, which further confirms the successful encapsulation of EP@SiO_2@TA-Cu bilayer shell in self-healing microcapsules.

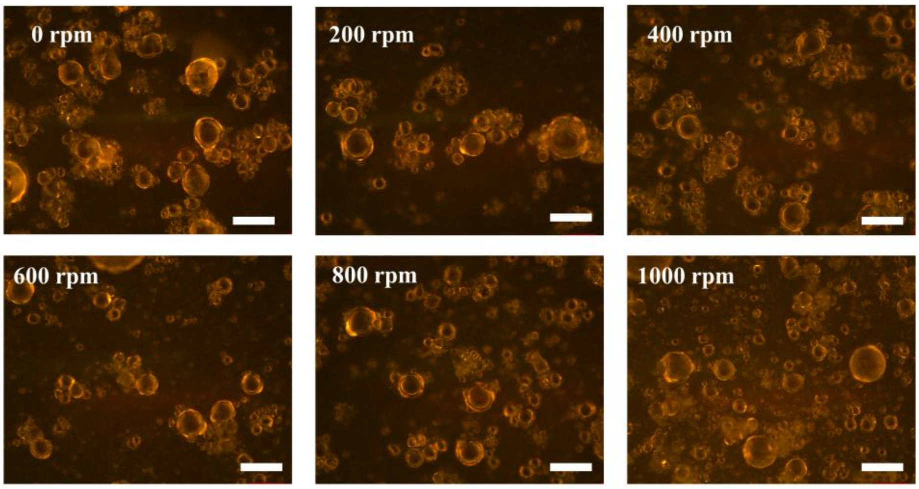

Figure S5. POM images of self-healing microcapsules in Stirring tests.

scale bar: 100 μm

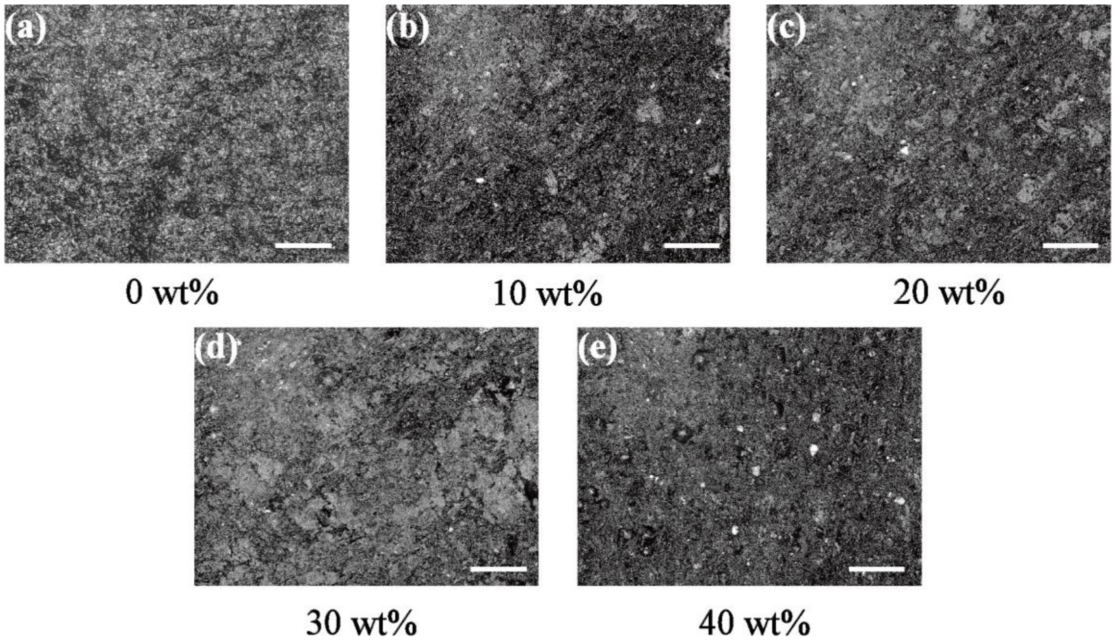

Figure S6. laser confocal microscopy images of coatings with different microencapsulates additions

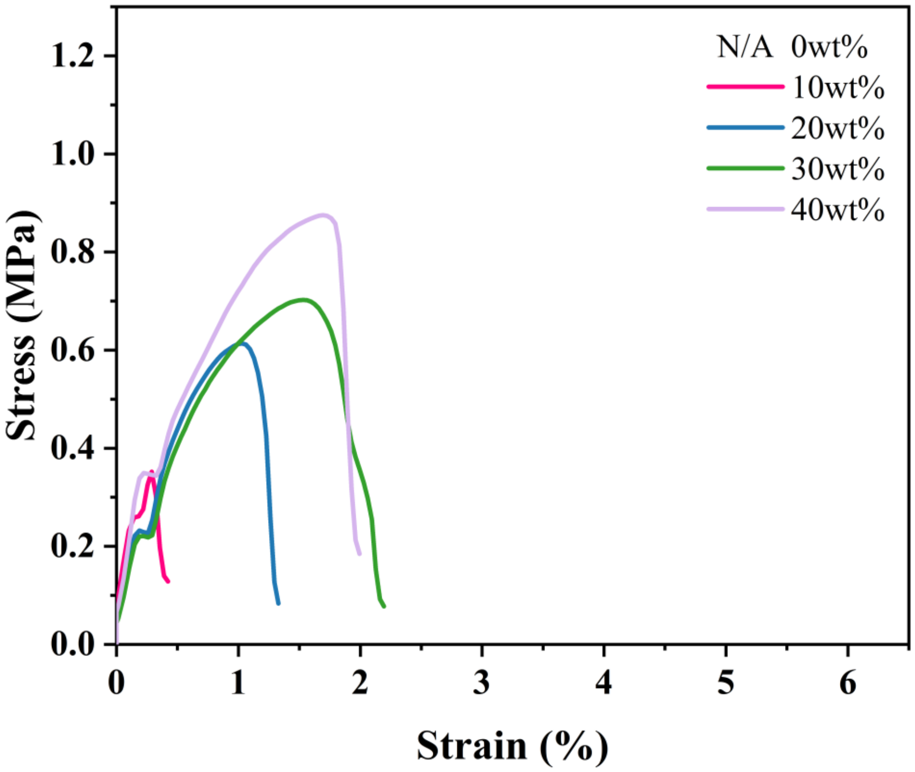

Figure S7. The tensile strength of the cut off coating after self-healings

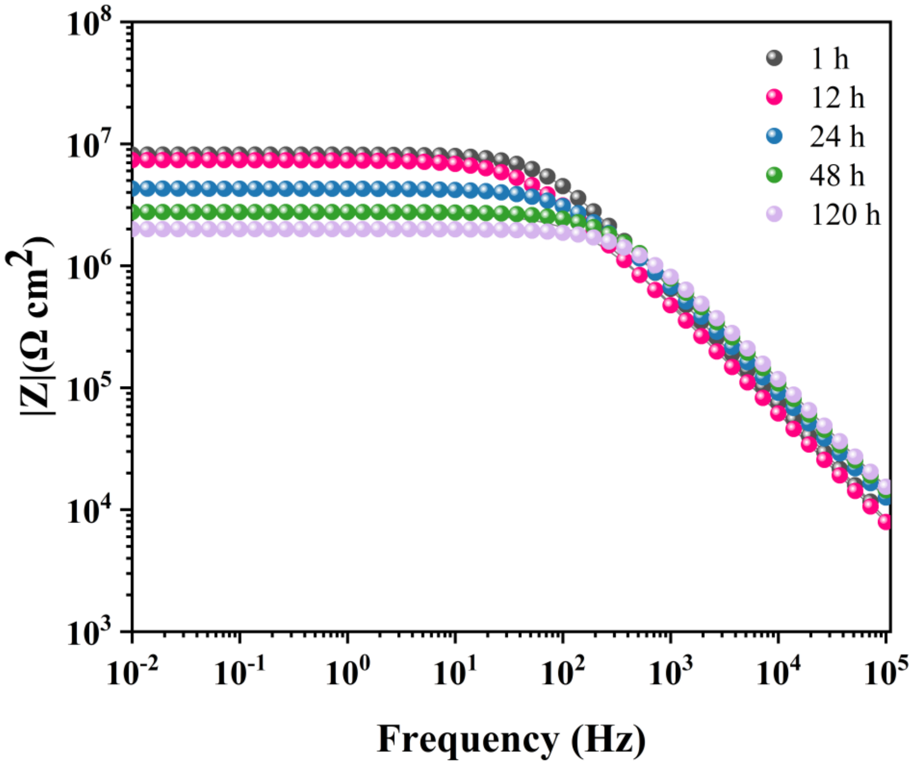

Figure S8. Bode plots of self-healing coatings with different times immersion in 3.5 wt % NaCl solution

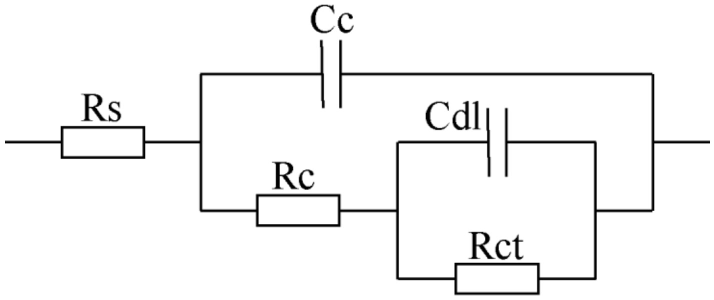

Figure S9. electrical equivalent circuits used to fit the impedance data
